# An Activating Janus Kinase-3 Mutation Is Associated with Cytotoxic T Lymphocyte Antigen-4-Dependent Immune Dysregulation Syndrome

**DOI:** 10.3389/fimmu.2017.01824

**Published:** 2017-12-15

**Authors:** Heiko Sic, Matthaios Speletas, Vanessa Cornacchione, Maximillian Seidl, Martin Beibel, Bolan Linghu, Fan Yang, Eirini Sevdali, Anastasios E. Germenis, Edward J. Oakeley, Eric Vangrevelinghe, Andreas W. Sailer, Elisabetta Traggiai, Hermann Gram, Hermann Eibel

**Affiliations:** ^1^Novartis Institute for Biomedical Research, Basel, Switzerland; ^2^Department of Immunology and Histocompatibility, Faculty of Medicine, School of Health Sciences, University of Thessaly, Biopolis, Larissa, Greece; ^3^Institute for Surgical Pathology, University Medical Center Freiburg, Freiburg, Germany; ^4^Center for Chronic Immunodeficiency, University Medical Center Freiburg, Freiburg, Germany; ^5^Novartis Institutes for Biomedical Research, Cambridge, MA, United States

**Keywords:** cytotoxic T lymphocyte antigen-4, janus kinase-3, haploinsufficiency, immune dysregulation syndrome, T regulatory cell, T follicular helper cell, common variable immunodeficiency, immune checkpoint

## Abstract

Heterozygous mutations in the cytotoxic T lymphocyte antigen-4 (CTLA-4) are associated with lymphadenopathy, autoimmunity, immune dysregulation, and hypogammaglobulinemia in about 70% of the carriers. So far, the incomplete penetrance of CTLA-4 haploinsufficiency has been attributed to unknown genetic modifiers, epigenetic changes, or environmental effects. We sought to identify potential genetic modifiers in a family with differential clinical penetrance of CTLA-4 haploinsufficiency. Here, we report on a rare heterozygous gain-of-function mutation in Janus kinase-3 (JAK3) (p.R840C), which is associated with the clinical manifestation of CTLA-4 haploinsufficiency in a patient carrying a novel loss-of-function mutation in CTLA-4 (p.Y139C). While the asymptomatic parents carry either the CTLA-4 mutation or the JAK3 variant, their son has inherited both heterozygous mutations and suffers from hypogammaglobulinemia combined with autoimmunity and lymphoid hyperplasia. Although the patient’s lymph node and spleen contained many hyperplastic germinal centers with follicular helper T (T_FH_) cells and immunoglobulin (Ig) G-positive B cells, plasma cell, and memory B cell development was impaired. CXCR5^+^PD-1^+^TIGIT^+^ T_FH_ cells contributed to a large part of circulating T cells, but they produced only very low amounts of interleukin (IL)-4, IL-10, and IL-21 required for the development of memory B cells and plasma cells. We, therefore, suggest that the combination of the loss-of-function mutation in CTLA-4 with the gain-of-function mutation in JAK3 directs the differentiation of CD4 T cells into dysfunctional T_FH_ cells supporting the development of lymphadenopathy, hypogammaglobulinemia, and immunodeficiency. Thus, the combination of rare genetic heterozygous variants that remain clinically unnoticed individually may lead to T cell hyperactivity, impaired memory B cell, and plasma cell development resulting finally in combined immunodeficiency.

## Introduction

Common variable immunodeficiency (CVID) is the most common primary immunodeficiency. It combines a variety of defects sharing low levels of IgG and IgA (1) and although a number of genetic defects has been found to be associated with CVID, the pathogenetic mechanisms of most of the cases remain to be determined (2, 3). Paradoxically, a large number of CVID patients suffer from complex autoimmunity and immune dysregulation syndromes (4) and some of these patients have mutations in *CTLA4* (5–7). Cytotoxic T lymphocyte antigen-4 (CTLA-4) is expressed by activated T cells and T regulatory (T_REG_) cells and acts as major negative regulator of T cell responses (8). Thus, the immune dysregulation syndrome is thought to be caused by the accumulation of hyperactive T cells. Genetic inactivation of both *Ctla4* alleles in mice causes lymphoproliferation, T cell infiltration into tissues, and leads to strongly increased serum antibody concentrations whereas inactivation of only one *Ctla4* allele (haploinsufficiency) is asymptomatic (9). The situation is more complex in humans because the same heterozygous *CTLA4* mutations can be found in patients with immunodysregulation syndrome and in asymptomatic carriers, who may only show subtle immunophenotypic changes like the expansion of FOXP3^+^ T_REG_ cells (7).

So far, epigenetic, environmental, and genetic factors have been discussed as potential cause for the incomplete penetrance of CTLA-4 mutations (6, 7), but it remained unclear how these factors may contribute to the development of a CTLA4-dependent immune dysregulation syndrome.

Here, we report that the combination of a rare heterozygous gain-of-function mutation in the Janus kinase-3 (*JAK3*) gene with a novel, heterozygous loss-of-function mutation in CTLA-4 severely affects the function of T_FH_ cells. The combination of both mutations correlates with immunodeficiency associated with lymphadenopathy, autoimmunity, and hypogammaglobulinemia, whereas carriers of either the CTLA-4 or the JAK3 single mutations are healthy. Residing in the highly conserved ligand binding motif, the CTLA-4 mutation (p.Y139C) abolishes the CD80/CD86 ligand binding of CTLA-4. The activating mutation in JAK3 (R840C, rs200077579) resides in the N-lobe of its kinase domain. JAK3 is mainly expressed in cells of the immune system and associates with the common γ-chain of cytokine receptors. Binding of the interleukins (IL)-2, IL-7, IL-9, IL-15, and IL-21 to their respective receptors activates JAK3, and by phosphorylating signal transducer and activator of transcription (STAT) transcription factors, the kinase regulates proliferation and cellular differentiation (10). While activating *JAK3* mutations have so far been associated with lymphoid hyperplasia and leukemia (11), loss-of-function and hypomorphic mutations lead to a broader variety of clinical phenotypes ranging from lymphoproliferative disorders and milder/later onset of immunodeficiency to severe combined immunodeficiency (12, 13). Activating JAK3 mutations, however, have not yet been reported in the context of primary immunodeficiencies.

## Materials and Methods

Additional information on Section “Materials and Methods” including the preparation of DNA libraries, sequencing, and antibodies used in flow cytometry, immunohistochemistry, and western blotting can be found in the Supplementary Material.

### Ethics Approval

Human peripheral blood was obtained from healthy donors and patient. Spleen and lymph node (LN) samples were obtained from patients undergoing splenectomy and LN biopsy. All material was used after written informed consent and in accordance with the approvals 78/2001 and 251/13 of the University Medical Center ethics committee.

### Histology

Formalin-fixed lymphoid tissues embedded in paraffin were cut into 3 µm slices and stained after deparaffinization and antigen retrieval with different antibodies as listed in supplementary methods. After applying horse radish peroxidase-coupled secondary antibodies, a brown chromogen reaction developed in the presence of DAB as substrate (Dako Autostainer Link^®^, Dako). Images were taken with a Zeiss Axioobserver using ZEN software. Colors were adjusted with Adobe Photoshop.

### Patient

The 23-year-old male patient had bronchiolitis during infancy and suffered from recurrent upper respiratory tract infections and chronic sinusitis during the last 4 years before the diagnosis of CVID. He was also treated for hypothyroidism the last 3 years before diagnosis. One year before the diagnosis of CVID, splenomegaly, generalized lymphadenopathy in the mediastinum and retroperitoneum, pancytopenia, and severe hypogammaglobulinemia were discovered in the course of gastroenteritis. The patient did not respond to vaccination with pneumococcal polysaccharides. His bone marrow was hypercellular and a stomach biopsy indicated lymphocytic infiltrates. Severe splenomegaly was treated with splenectomy; LN biopsies did not reveal malignancies. The family members are healthy with normal serum immunoglobulin levels except for the Hashimoto’s thyroiditis of the mother.

### Sequencing Analysis of CTLA-4 and JAK-3

Genomic DNA was extracted from peripheral blood using the QIAamp DNA Blood Mini Kit (Qiagen, UK). All exons and exon-intron boundaries of *CTLA4* and *JAK3* genes were amplified using primers shown in Tables 1 and 2. PCR products were purified (Qiagen) and sequenced using an ABI Prism 310 genetic analyzer (Applied Biosystems, Foster City, CA, USA) and a BigDye Terminator DNA sequencing kit (Applied Biosystems).

**Table 1:** The sequences of primers used for the amplification of *CTLA4*.

Exon	Primers	Sequence
Exon 1	Forward	5′-TTCAAgTgCCTTCTgTgTgTg-3′
	Reverse	5′-AATCACTgCCτTTgACTgCT-3′
Exon 2	Forward	5′-gAgAggggAAggggTAAgTg-3′
	Reverse	5′-AgACTgCAATgCAACAggTg-3′
Exon 3	Forward	5′-TATTggTgggCTACCCATgC-3′
	Reverse	5′-CCCTgCTCAgAAgCACATgA-3′
Exon 4	Forward	5′-TggCTTCCgTATTCCTCAgT-3′
	Reverse	5′-CTCCCTgCCTTTTCCTTCTT-3′

**Table 2:** The sequences of primers used for the amplification of *JAK3*.

Exon	Primers	Sequence
Exon 2 (including start codon)	Forward	5′-CATgCCCTCCCTgCTCAgAA-3
	Reverse	5′-AAgCCAACCCTgCACACCCTT-3′
Exons 3–5	Forward	5′-gATgCTggCACTCCTgAAggg-3′
	Reverse	5′-ggAgAgggCTgggTTCgTg-3′
Exon 6	Forward	5′-CTgTggggTCCCTgTCCgA-3′
	Reverse	5′-CgCTCAgCCCAACCCTTCACT-3′
Exons 7–8	Forward	5′-CggCTTggAAgggTTgAATgg-3′
	Reverse	5′-CTgTgCggCAggTgTggTT-3′
Exons 9–10	Forward	5′-ggTgTCACCTggCAAggAT-3′
	Reverse	5′-AACTTCCTgAgCCAACAAATC-3′
Exons 11–12	Forward	5′-AAAgCCATgTgCCCTgAAgTCT-3′
	Reverse	5′-CgCCCAggTCCCTgTgTgT-3′
Exon 13	Forward	5′-CCCgTATCAgAAAATCATggTA-3′
	Reverse	5′-CCTAgACTCCCAACCAATgAAA-3′
Exon 14	Forward	5′-TTCCAggCATTCCAggCAAAT-3′
	Reverse	5′-CACTCCCAATTCCTCTTCCACC-3′
Exon 15–17	Forward	5′-gTCAggAgTCAgggACgATgCT-3′
	Reverse	5′-CCCCTCCAACCTCACCAgAC-3′
Exons 18–19	Forward	5′-TTTgCCTgggACAgAgTgg-3′
	Reverse	5′-CTggCAggAgggTAAgAATgT-3′
Exons 20–21	Forward	5′-gTCATTgTTgCggTTCCCATA-3′
	Reverse	5′-CgggAgACAgAggAgCCAgTg-3′
Exons 22–23	Forward	5′-TggAgACgggACTgACCTgCT-3′
	Reverse	5′-CCTCATCggCCTCACACTCTA-3′
Exon 24	Forward	5′-TgggCAACAAgAgCgAAACTT-3′
	Reverse	5′-TTTgggCCAggACTCAgAg-3′

### Flow Cytometry

Cells were analyzed by flow cytometry as described before (14, 15). FOXP3 expression was tested using either the FOXP3 staining kit (eBioscience) following the manufacturer’s instructions, or Lyse/Fix-buffer (BD, 20 min, 37°C) and Methanol (90%, 1 h, −20°C) followed by staining for intracellular antigens. Analyses were performed using a Canto II or LSRFortessa (BD) flow cytometer and Diva (BD) and FlowJo software (TreeStar).

### Cell Based *In Vitro* Assays

PBMC and EBV lines were isolated and cultivated and tested for mycoplasma contamination as described before (14). Jurkat and HEK293T/17 cells were obtained from ATCC (Manassas, VA, USA). The acute megakaryoblastic leukemia line M07e was received from DSMZ (Braunschweig, Germany). The cell culture medium for MO7e was supplemented with 10 ng/ml GM-CSF (R&D Systems). Dose-dependent proliferation of transduced MO7e cells (5 × 10^4^ cells/ml) was monitored by flow cytometry using 50–300 U/ml IL-2 (Novartis) or 10 ng/ml GM-CSF.

Activation of T cells was performed with 0.3–1 × 10^6^ PBMCs by adding anti-CD3 (1 µg/ml, OKT-3, Novartis, Basel, Switzerland) and anti-CD28 (1 µg/ml, 15E8, Novartis) in complete IMDM.

Suppression assays with Jurkat T cells were performed by mixing 5 × 10^4^ naïve CD4 T cells enriched from PBMCs using the EasySep human (naïve) CD4^+^ T Cell isolation kit (Stemcell Technologies, Vancouver, BC, Canada), with 1 × 10^4^ irradiated (50 Gy) EBV cells, increasing numbers of transduced, YFP sorted, and irradiated (50 Gy) Jurkat cells. The CD4 T cells were activated with anti-CD3 (1 µg/ml), cultured in complete IMDM and pulsed with 1 μCi/well of ^3^H-Thymidine (Perkin Elmer, Norwalk, CT, USA) for 16 h. The incorporation of ^3^H-Thymidine was measured using a β-plate counter (Wallac).

T_FH_/B cell co-culture assays were carried out with PBMCs sorted by FACS into CD19^+^CD27^−^IgA^−^IgG- (naïve) B cells and CD4^+^CD8^−^CD45RA^−^CXCR5^+^ T_FH_ cells. The purity of the sorted B and T cell subsets was >95%. 1 × 10^4^ T_FH_ cells/well were co-cultured with allogeneic B cells (1:1) and activated with endotoxin-reduced Staphylococcal enterotoxin B (SEB, 1 µg/ml, Toxin Technology, Sarasota, FL, USA) in complete RPMI 1640 medium complemented with 2-mercaptoethanol and in 384 well plates. After 7 days, supernatants were collected and levels of secreted cytokines were assessed using Meso Scale Discovery (Meso Scale Diagnostics, LLC, MD, USA) following the manufacturer’s instructions and an MSD Sector Imager 6000 (MSD).

Intracellular cytokines expressed in activated T_FH_ cells from the co-culture assay were detected upon activating the T cells for 6 h with 100 ng/ml PMA (Sigma-Aldrich, St. Louis, MO, USA) and 750 ng/ml ionomycin (Sigma-Aldrich) in the presence of Brefeldin A (10 mg/ml, Sigma-Aldrich), which was added after 2 h.

CD19^+^CD27^−^IgA^−^IgG^−^ (naïve) B cells were differentiates *in vitro* into plasmablasts in U96 well plates with 10^4^/well in the presence of CD40L (1 µg/ml, Novartis), IL-4 (20 ng/ml, R&D), and IL-21 (20 ng/ml, Novartis) in complete RPMI 1640 medium supplemented with Glutathione (Sigma-Aldrich), MEM-non essential amino acids (Invitrogen), and insulin–transferrin-selenium-x supplement (Invitrogen). Cells and supernatant were collected after 6 days and tested for the expression of surface markers by flow cytometry and for the secretion of IgM and IgG by ELISA as described before (14–16).

### Binding Assays

EBV cells (5 × 10^5^) were incubated with abatacept (orenica, Bristol-Myers Squibb, New York, NY, USA) or recombinant CTLA-4-Ig (Novartis). CD28 on Jurkat cells (5 × 10^5^) was first blocked by treatment with anti-CD28 (CD28.2, BD) followed by incubation with recombinant CD80/CD86 (R&D). The cells were then stained with FITC-coupled anti-human IgG (eBioscience) and analyzed by flow cytometry.

### SDS-PAGE and Western Blotting

Western blotting was performed as described before (15). Transduced MO7e cells were washed twice with FCS-free medium and incubated overnight in IMDM, 0.1% FCS. Then, 7 × 10^5^ cells were washed with ice-cold PBS, lysed with NuPAGE LDS sample buffer (5 × 10^6^ cells/ml), vortexed, and heated to 70°C for 10 min. 20 µl/sample were separated by 4–12% PAGE and transferred to PVDF membranes using the Trans-Blot turbo system (Bio-Rad, CA, USA).

### Expression Plasmids

Synthetic 738 bp DNA fragments encoding either wild-type or Y139C mutant CTLA-4 were obtained from Integrated DNA Technologies (Leuven, Belgium). The ssBMS-CTLA-4-Ig expression plasmid and the pcDNA3.1-based plasmids containing JAK3-WT or JAK3-A572V were obtained from C. Haan (17). CTLA-4-Y139C and JAK3-R840C were generated by using the QuikChange XL site-directed mutagenesis kit (Agilent Technologies) and following oligonucleotides: CTLA-4 (full-length)

f-5′-CATGTACCCACCGCCATGCTACCTGGGCATAG-3′ and
r-5′-CTATGCCCAGGTAGCATGGCGGTGGGTACATG-3′;
CTLA-4 (ssBMS)
f-5′-GATGTACCCCCCTCCCTGCTACCTGGGCATCG-3′, r-5′CGATGCCCAGGTAGCAGGGAGGGGGGTACATC-3′;
JAK3:
f-5′-CGTGGAGCTGTGCTGCTATGACCCGCTAGG-3′ and
r-5′-CCTAGCGGGTCATAGCAGCACAGCTCCACG-3′.

The lentiviral expression vectors pNL-CEF-eGFP and pNL-CEF-YFP/CFP were described before (14, 16). CTLA-4 coding regions were cloned into pNL-CEF-YFP/CFP by replacing a 702 bp fragment with the corresponding synthetic DNA fragments. An internal ribosomal entry site (IRES) was cloned from pIRES2-eGFP (Clontech Laboratories, Mountain View, CA, USA) into pNL-CEF-eGFP generating the pNL-CEF-IRES-eGFP backbone. JAK3 coding regions were then sub-cloned into pNL-CEF-IRES-eGFP. Enzymes used for cloning were from New England Biolabs (Ipswich, MA, USA), Roche (Basel, Switzerland) or from Fermentas (Vilnius, Lithuania). All constructs were verified by Sanger sequencing.

### Transfections and Infections

Lentiviral particles were generated using a standard protocol (14) upon transfection of HEK293T/17 cells with pCDNLBH* and pVSV-G, and the pNL-based constructs. Supernatants were collected 2 days after transfection, filtered (0.45 µm, Pall Corporation, NY, USA) and 1 ml were used to spin-infect 5 × 10^5^ Jurkat, MO7e cells, or anti-CD3 (1 µg/ml) and anti-CD28 (1 µg/ml) activated PBMCs or CD4^+^ T cells.

### Recombinant Protein Production

Recombinant CTLA-4-Ig was produced by growing suspension-adapted HKB11 cells (Human Embryonic Kidney cells fused with Burkitt’s lymphoma cells, Bayer) transfected with expression plasmids in serum-free culture medium M11V3 in a WAVE bioreactor. Cultures were supplemented with an equal volume of DM133 enriched with Yeastolate (2 g/l) (Irvine Scientific, Santa Ana, CA, USA) and incubated for 3–8 days.

Supernatants were filtered through a dead end filter (Millistak + Pod Disposable Depth Filter System Merck Millipore, Darmstadt, Germany), sterilized (0.22 µm stericup filter, Millipore), and concentrated (Cross flow filter Hemoflow, 10 kDa, Fresenius, Bad Homburg, Germany). Proteins were purified by MabSelect affinity chromatography (GE Heathcare, Chicago, IL, USA), eluted (50 mM Tris, 90 mM NaCl pH 3.2) and immediately neutralized to pH 7.3 by slow addition of 1 M Tris pH 10.

### Protein Modeling

The modeling of the JAK3 JH2-JH1 homology domain was done using TYK2 (PDB 4OLI) (11) as template and Prime 4.2 software of Schrödinger Release 2015-4 (Schrödinger, New York, NY, USA). Images of the CTLA-4/CD80 structure [PDB 1I8L (18)] and the JAK3 model were generated with ICM (Molsoft LLC, San Diego, CA, USA).

### Statistical Analysis

Significant differences were analyzed by unpaired Student’s *t*-test, by Tukey’s or Holm–Sidak’s (one-way ANOVA) or Dunnett’s or Holm–Sidak’s (one-way ANOVA) multiple comparisons test using GraphPad Prism software (GraphPad). Values of *P* ≤ 0.05 were considered significant.

## Results

### A Novel Loss-of-Function Mutation in *CTLA4*

**Figure 1:**
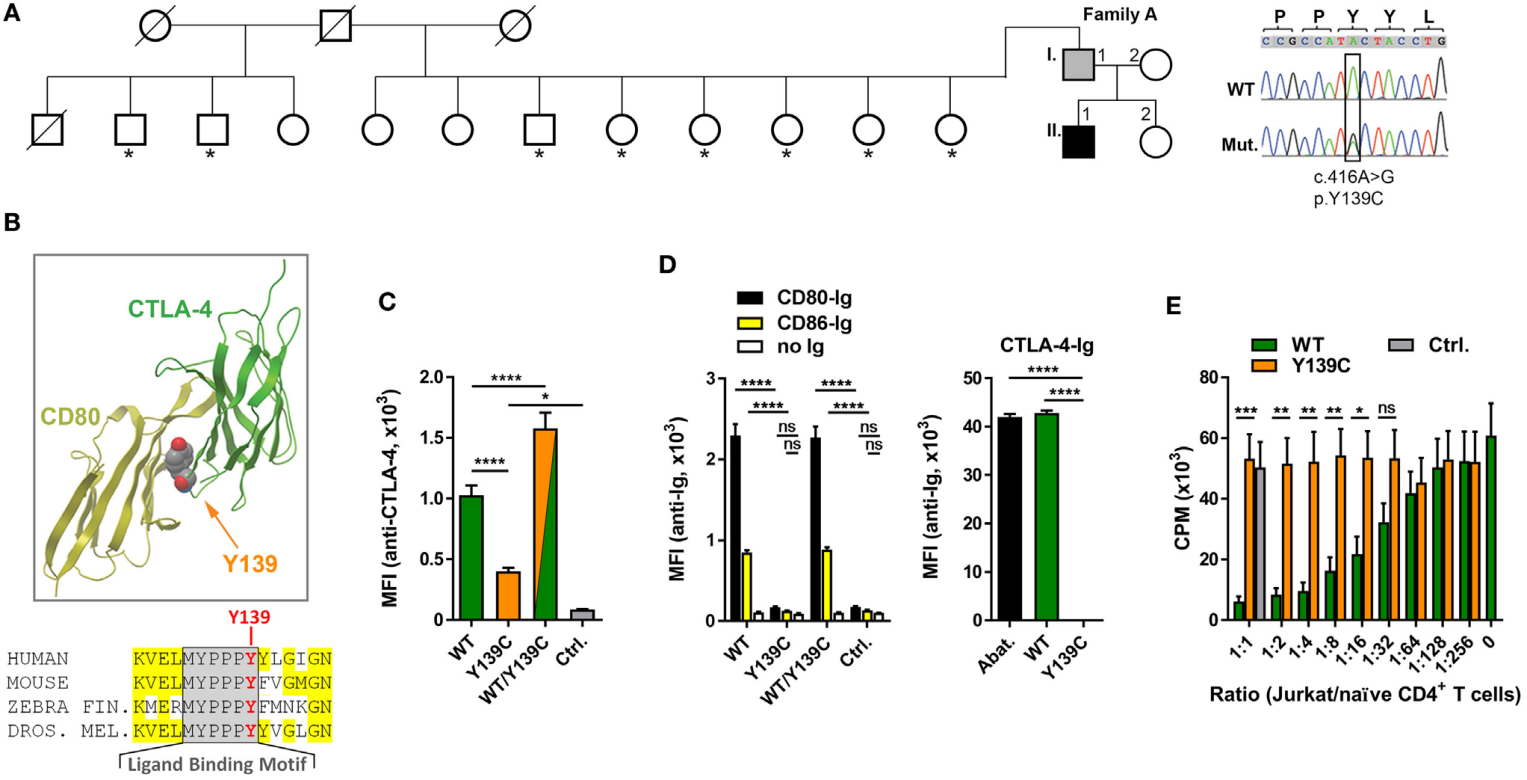
A novel mutation in cytotoxic T lymphocyte antigen-4 (CTLA-4) ligand binding motif. **(A)** Pedigree of family A, and Sanger sequencing of the mutation. Squares: male subjects; circles: female subjects; black filled symbol: patient with *CTLA4* mutation; gray filled symbol: *CTLA4* mutation carrier; crossed-out symbols: deceased subjects. *CTLA4* was sequenced in all individuals of whom genomic DNA was available (asterisks). The position of the missense mutation (Mut.) in the cDNA (c.416A>G) is boxed and the resulting amino acid change p.Y139C is shown. WT, wild type. **(B)** Residue Y139 is highly conserved in the CTLA-4 MYPPPY binding motif as shown by the sequence comparison. Structure of CTLA-4 (green ribbon, right) and interaction-site of Y139 with CD80 (yellow ribbon, left). Position of Y139 is shown as space-filling model. **(C)** Mean fluorescence intensities (MFI) of CTLA-4 signals from Jurkat T cells expressing wild-type (WT, green bar), or mutant (Y139C, orange bar), or both forms (WT/Y139C) of CTLA-4 stained with anti-CTLA-4 antibodies and analyzed by flow cytometry. The cells express CTLA-4 proteins fused at their C-terminus with YFP (WT) or CFP (Y139C); Ctrl.: non-transduced cells. **(D)** Left bar graph: MFI of Jurkat T cells expressing WT, or Y139C mutant, or both forms of CTLA-4 stained with recombinant CD80-Ig (black) or CD86-Ig (yellow) and analyzed by flow cytometry. Right bar graph: MFI of EBV cells expressing CD80 and CD86 stained with abatacept (Abat., black), wild type (WT, green), or mutant CTLA-4-Ig and analyzed by flow cytometry. **(E)** *In vitro* proliferation assay with naïve CD4 T cells activated with anti-CD3 in presence of heterologous EBV cells. T cell proliferation was inhibited by adding increasing numbers of Jurkat T cells expressing WT CTLA-4 (WT, orange bars) but not by cells expressing Y139C mutant CTLA-4 (green bars) (Ctrl.: Jurkat T cells). Data in **(C,D)** are from three experiments done in triplicates and in **(E)** from three experiments with five different healthy donors shown as mean ± SEM. ****P ≤ 0.0001; ***P < 0.001; **P < 0.01; *P ≤ 0.05 [one-way ANOVA, Tukey’s **(C)** and two-way ANOVA, Holm–Sidak’s **(D,E)** multiple comparisons test].

Analyzing the exomes of a primary antibody deficiency cohort of 37 individuals from 9 families, we identified in one family a novel heterozygous missense mutation (p.Y139C) in the *CTLA4* gene (Figure 1A). The mutation is shared by the patient (A.II.1) and the father (A.I.1). While the patient is diagnosed with hypogammaglobulinemia, signs of autoimmunity and severe lymphoproliferation which required splenectomy, the father is asymptomatic and has normal IgG and IgA levels (Table 3). Since the p.Y139C CTLA-4 variant has not been documented before and because it is not shared by any of A.I.1 siblings, it most likely has arisen *de novo* in A.I.1. The mutation is localized in the evolutionary highly conserved ligand-binding motif (MYPPPY) of CTLA-4 and changes the second tyrosine to cysteine (Figure 1B). A very similar change (Y139A) introduced by site-directed mutagenesis was shown to abolish CTLA-4 binding to CD80 and CD86 (19, 20).

**Table 3:** Laboratory features.

Laboratory												
	Normal values		A.I.1 (father)		A.II.1 (patient)							
			1-2015		7-2011		3-2012^a^		7-2013		7-2015	

	Cells/μL	(%)	Cells/μL	(%)	Cells/μL	(%)	Cells/μL	(%)	Cells/μL	(%)	Cells/μL	(%)
WBC	4,500–10,500		9,300		3,600		7,300		11,600		13,700	
Neutrophils	1,500–6,500		5,840		1,569		4,416		6,610		8,640	
Monocytes	200–1,000		530		324		613		1,260		1,099	
Lymphocytes	1,200–3,800		2,511		1,707		2,270		2,571		1,916	
CD3^+^	700–2,100	(55–83)	1,404	(55.9)	1,512	**(88.6)**	1,580	(69.6)	1,923	(74.8)	1,412	(73.7)
CD3^+^CD4^+^	300–1,400	(28–57)	467	(33.3)	1,123	**(74.3)**	980	**(62)**	1,053	(55)	747	(39)
CD3^+^CD8^+^	200–900	(10–39)	236	(16.8)	316	(20.9)	515	(32.6)	638	(33.3)	619	(32.3)
CD4/CD8	1–3.6		1.98		3.6		1.9		1.65		1.21	
CD16^+^CD56^+^	90–600	(7–31)	**856**	**(34.1)**	**56**	**(33.3)**	547	(24.1)	177	**(6.9)**	**98**	**(5.1)**
CD19^+^	100–500	(6–19)	216	(8.6)	137	(8)	143	(6.3)	468	(18.2)	356	(18.6)
**Immunoglobulin (mg/dl)**	**A.I.1 (father)**	**A.II.1 (patient)**										
IgG (847–1,690)	1,120	**387**										
IgM (64–249)	102	57.9										
IgA (99–300)	266	**47.5**										
RF	Negative	Negative										
ANA	Negative	Negative										

*^a^Patient already subjected to splenectomy and receiving Ig replacement bold out of normal range*.

To address the question if the Y139C mutation changes the surface expression of CTLA-4, we constructed lentiviral expression vectors for wild-type (WT) and mutant (Y139Y) CTLA-4 expressing fusion proteins of CTLA-4 linked at the C-terminal end to CFP and YFP.

Co-expression of wild-type and mutant forms revealed that the mutant did not interfere with the expression of the wild-type form. However, comparing the expression of both fusion proteins, we found that the Y139C mutant was expressed at lower levels on the cell surface (Figure 1C) although total expression levels of CTLA-4-YFP and CTLA-4-Y139Y-YFP were very similar (Figure S1 in Supplementary Material).

In contrast to CTLA-4-WT, CTLA-4-Y139C did not bind recombinant soluble CD80 or CD86. *Vice versa*, recombinant CTLA-4-Y139C did not bind to CD80/CD86 expressed on an EBV immortalized B cell line (Figure 1D; Figure S2 in Supplementary Material). In an *in vitro* suppression assay, Jurkat T cells expressing the CTLA-4-Y139C variant did not suppress co-stimulation-dependent proliferation of naïve CD4^+^ T cells (Figure 1E). Analyzing the T_REG_ cells of the patient, we found that they did not suppress the proliferation of activated responder T cells *in vitro*, whereas the T_REG_ cells of the carrier A.I.1 still showed suppressor activity under the assay conditions (Figure S3 in Supplementary Material). We, therefore, conclude that CTLA-4-Y139C is a loss-of-function mutation with incomplete penetrance *in vivo* and similar to other previously reported mutations inactivating CTLA-4 (5–7).

**Figure 2:**
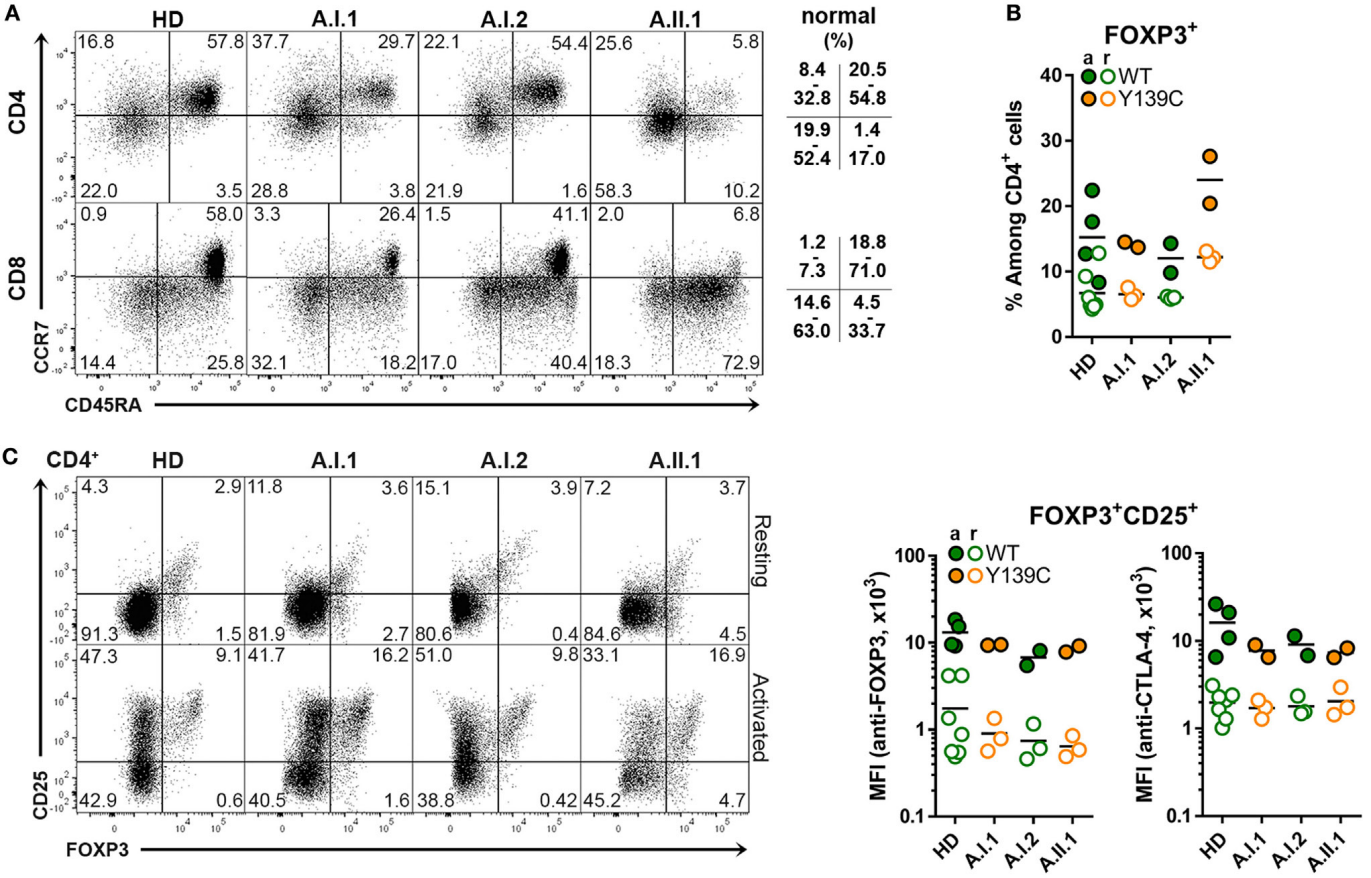
Cytotoxic T lymphocyte antigen-4 (CTLA-4) expression in T_REG_ cells. **(A)** Representative flow cytometry plots of PBMCs stained with anti-CD3, CD4, CD8 CD45RA, and CCR7 antibodies. The plots show the population frequencies of naïve T cells (upper right), central memory T cells (upper left), effector memory T cells (bottom left) and CD45RA^+^ effector memory T cells (bottom right) cells in the CD3^+^CD4^+^ or CD3^+^CD8^+^ T cell subsets isolated from a healthy donors (HD), family members A.I1.1, A.I.2, and patient A.II.1. The normal range of each subset is indicated. **(B)** Percentage of FOXP3^+^ CD4^+^ T_REG_ cells from HD and from family A members A.I.1, A.I.2, A.II.1 (patient). **(C)** Representative flow cytometry plots showing T_REG_ cells of resting and anti-CD3 + anti-CD28-activated CD4^+^ T cells stained with anti-CD25 and anti-FOXP3 antibodies. The bar graph displays the mean fluorescence intensity of FOXP3 and of CD25 signals of resting (open circles) and activated (filled circles) CD4^+^ T cells isolated from HD and family A members A.I.1, A.I.2, and A.II.1 (patient). Horizontal lines represent mean values. The dots in **(B,C)** represent the samples of individual HD or family member analyzed in replicates in repeated experiments carried out after different visits.

The percentage of CD45RA^+^CCR7^+^ naïve T cells was reduced in the patient A.II.1 but not the healthy carrier A.I.1, while the proportion of CD45RA^–^CCR7^–^ CD4^+^ effector memory T cells (T_EM_ cells) and of CD45RA^+^CCR7^–^ CD8^+^ effector memory T cells (T_EMRA_ cells) was markedly higher than in controls (Figure 2A). Similar changes in the T cell compartment have been reported previously for other CTLA-4 haploinsufficiency patients (6, 7), including an increased percentage of CD4^+^FOXP3^+^ T cells, which was higher in patient A.II.1 than in the carrier A.I.1 (Figure 2B). In contrast to the increase in CD4^+^FOXP3^+^ T cells frequency, the expression levels of intracellular FOXP3 and CTLA-4 in FOXP3^+^CD25^+^ T_REG_ cells were comparable among all family members and healthy donors (Figure 2C). Therefore, we conclude that CTLA-4 haploinsufficiency in A.I.1 and A.II.1 results—like the recently described CTLA-4-P137R mutation (21)—from a functional defect of CTLA-4 and not from impaired expression of CTLA-4 by T_REG_ cells.

### A Rare Gain-of-Function Mutation in *JAK3*

**Figure 3:**
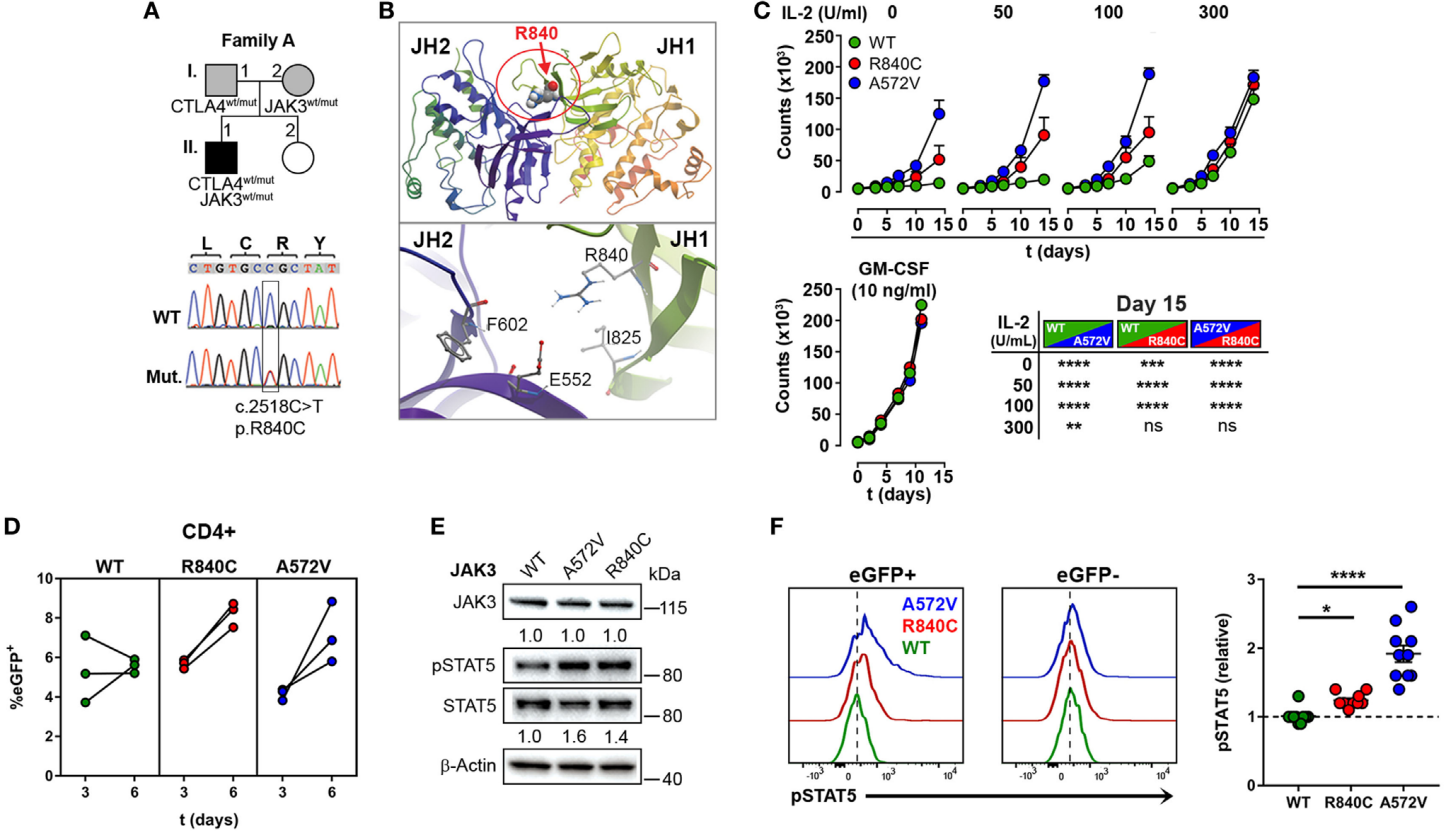
A rare gain-of-function mutation in Janus kinase-3 (JAK3). **(A)** Pedigree of family A showing inheritance of *CTLA4* and *JAK3* mutations and the confirmation of the JAK3 mutation by Sanger sequencing. Squares: male subjects; circles: female subjects; black-filled symbols: patient with mutation; gray filled symbols: mutation carriers. The position of the JAK3 mutation (Mut.) in cDNA is boxed and the nucleotide and amino acid changes are indicated as c. and p., respectively. WT, wild-type. **(B)** Structural model of the JAK3 Janus homology domains JH1 and JH2 domains. The position of residue R840 changed by the c.2518C missense mutation to Cysteine is represented as space-filling model and the N-lobe of the Kinase domain is circled (red). The lower panel shows the negatively charged R840 residue and its potential contacts with the positively charged E552 residue. **(C)** Growth curves of MO7e cells expressing wild type (WT, green color), R840C (red), or A572 (blue) JAK3 cultured w/o or with increasing concentrations of IL-2 or GM-CSF. Significant differences between the growth curves are shown in the lower right panel. Data represent mean ± SEM of three experiments. **(D)** Percentages of GFP^+^ primary human CD4^+^ T cells lentivirally transduced with WT (green), R840C (red), or A572V (blue) JAK3 constructs. Cells were activated first with anti-CD3 and anti-CD28 and cultivated from day 3 on in medium containing only IL-2 (2 U/ml). **(E)** Western blots showing JAK3 and STAT5 expression and STAT5 phosphorylation in MO7e cells expressing WT, A572V, or R840C JAK3 variants after activation with 50 U/ml IL-2 for 10 min. JAK3 expression is normalized to β-Actin; STAT5 phosphorylation is normalized to total STAT5 and compared to JAK3-WT. **(F)** Representative flow cytometry histograms showing basal STAT5 phosphorylation in CD4^+^ T cells transduced with JAK3 expression vectors (eGFP^+^) compared to the corresponding non-transduced (eGFP^−^) cells. Right panel: the plot shows relative pSTAT5 levels calculated for each sample as (MFI of pSTAT5 of eGFP^+^ cells)/(MFI of pSTAT5 of eGFP^−^ cells). Horizontal lines represent mean values. Dots in **(D,F)** represent individual healthy donor samples transduced with JAK3 expression constructs and are from three and five experiments. ****P ≤ 0.0001; ***P < 0.001; **P < 0.01; *P ≤ 0.05 [**(C)** two-way ANOVA, Tukey’s or **(F)** one-way ANOVA, Holm–Sidak’s multiple comparisons tests].

Since the heterozygous *CTLA4* mutation was found in the patient and in the healthy carrier, we assumed that the patient might have inherited another genetic defect with incomplete penetrance from his mother (A.I.2). This genetic defect would enhance the consequences of CTLA-4 haploinsufficiency and lead to an overt immune dysregulation syndrome. Therefore, we searched for rare genetic variants in the exome of patient A.II.1, which were inherited from the mother A.I.2, but not from the father A.I.1. A rare, heterozygous missense *JAK3* mutation (p.R840C, rs200077579) fulfilled these criteria. Among Europeans, missense mutation has been found in 1/3335 individuals (22, 23). Since the R840 residue is located in the N-lobe of the JH1 domain (24) close to a loop of a beta-sheet motif, which covers the ATP binding site, the R840C mutation might change the conformation of the N-lobe and its ability to interact with the pseudokinase domain (Figures 3A,B). This would change the kinase function and the activity of JAK3. As a consequence, cells expressing R840C JAK3 would respond differently to ILs like IL-2, which bind to receptors containing the JAK3-associated cytokine receptor common γ-chain.

Using different readout systems, we, therefore, tested the activity of the R840C variant in response to IL-2. First, we transduced the IL-2 indicator cell line MO7e with lentiviral expression vectors encoding the wild-type form of JAK3, the constitutive-active, and lymphoma-associated variant JAK3-A572V (17), or the JAK3-R840C variant, and analyzed IL-2-dependent growth at increasing IL-2 concentrations (Figure 3C). Different from wild-type JAK3, the constitutive-active A572V JAK3 and, to a lesser extent, the JAK3-R840C mutant supported IL-2-independent growth as well as higher growth rates at low IL-2 concentrations (Figure 3C). Similar to MO7e cells, expression of the JAK3 variant R840C also enhanced the growth of lentivirally transduced primary human CD4^+^ T cells (Figure 3D). Next, we assessed the phosphorylation of STAT5, the direct target of JAK3 kinase activity in MO7e (Figure 3E) and primary human CD4^+^ T cells (Figure 3F) transduced either with wild-type JAK3 or the gain-of function mutants of JAK3. As expected, basal level and IL-2-induced STAT5 phosphorylation was higher in cells expressing the JAK3-R840C or the constitutive active A572V variant than in cells expressing WT JAK3 (Figures 3E,F). Thus, the R840C missense mutation results in a gain-of-function variant of JAK3 as it increases the kinase activity of the enzyme.

Compared to the constitutive-active and lymphoma associated JAK3-A572V variant, the R840C variant has lower intrinsic JAK3 kinase activity, which explains why it has not yet been reported in conjunction with T cell hyperplasia, and why the carrier A.I.2 does not show signs of hematological malignancies.

### Germinal Center Hyperplasia and Changed Architecture of LNs and Spleen

**Figure 4:**
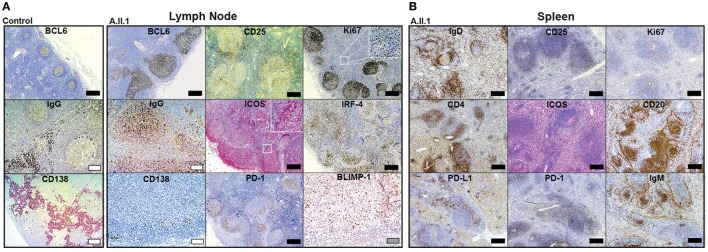
Immunohistochemistry of patient lymph node (LN) and spleen samples. Representative images of immunoperoxidase stainings of LN **(A)** and spleen **(B)** tissues. Large germinal centers were identified by staining for BCL6 in the patient’s LN and compared to LN of a healthy control (control). Additional GC markers, such as IgG defining immunoglobulin class-switched plasmablasts, Ki67 for labeling centroblasts, ICOS and PD-1 for follicular T-helper cells (T_FH_ cells), IRF-4 marking plasma blasts, and BLIMP-1 for GC B cells poised to develop into plasma cell are shown in **(A)**. Additional staining of IgD (resting follicular B cells), IgM (follicular and marginal zone B cells), CD20 (B cells), CD4 (T-helper cells), Ki67 (proliferating cells), ICOS and PD-1 (T_FH_ cells), PD-L1 (B cells and DCs), and CD25 (activated T cells and T_REG_ cells) in the spleen are shown in **(B)**. Mature plasma cells were identified by staining for CD138 in **(A)**. Scale bars; black, 500 µm; white, 100 µm; gray, 50 µm.

Our data revealed that the patient’s hypogammaglobulinemia is associated with CTLA-4 haploinsufficiency, increased JAK3 activity, and increased percentages of circulating CD4^+^ and CD8^+^ effector memory T cells. Since IgG and IgA-secreting long-lived plasma cells develop in germinal centers, we assumed that the immunological changes described above correlate with changes in the architecture of germinal centers. To this end, we analyzed paraffin-embedded tissue sections of spleen and LN samples of the patient using a set of markers defining T cell and B cell subsets. LN sections showed many follicles with very large, irregularly shaped BCL6-positive germinal centers (GC) containing many ICOS and PD-1 positive T_FH_ cells (Figure 4A). In spite of the GC hyperplasia, the typical polarization was clearly detected with distinct dark zones containing proliferating Ki67^+^ cells, and light zones containing IRF4^+^ GC B cells. Although the GCs contained IgG^+^ class-switched plasmablasts as well as BLIMP-1 expressing plasma cell precursors (Figure 4A), mature CD138^+^ plasma cells were not detected in GCs and found only in very small numbers in extrafollicular areas. Staining of B cells for IgD, IgM, and CD20 in spleen sections (Figure 4B) revealed a “follicle in the follicle-like” architecture in both LN and spleen. These structures also contained large ICOS^+^ and PD-1^+^ germinal centers with CD25^+^ cells, which include activated and T_REG_ cells. Therefore, the development of GC B cells into plasma cells seems to proceed up to the stage of plasmablasts within GCs but appears not to continue beyond this stage because CD138^+^ extrafollicular plasma cells were almost absent. Similar to the differentiation into plasma cells, the development of switched memory B cells was defective, as memory B cells were not detected in circulation (see below).

### High Frequency of PD-1 and TIGIT Expressing Circulating T_FH_ Cells

**Figure 5:**
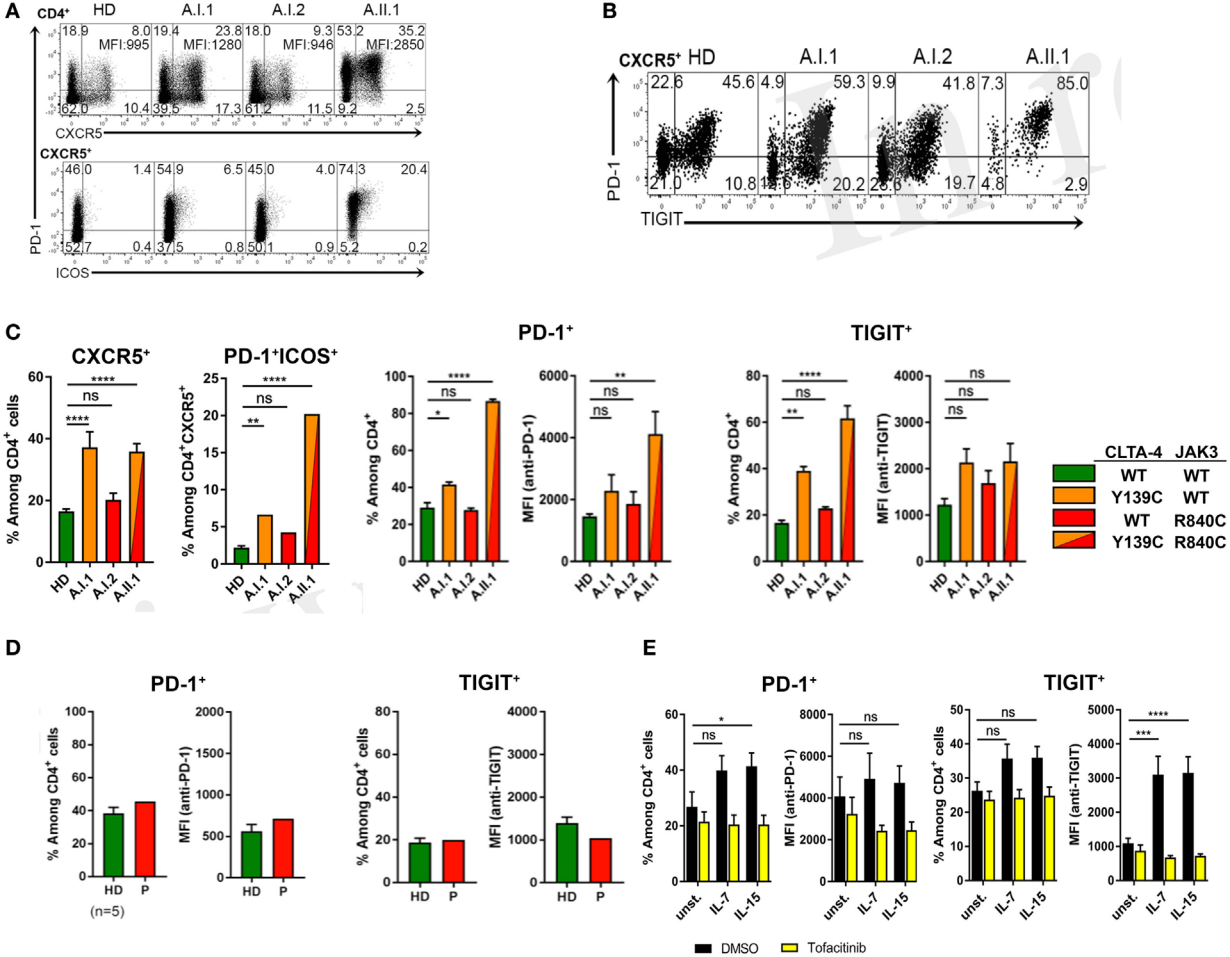
PD-1 and TIGIT expression by T_FH_ cells. **(A)** PD-1, CXCR5, and ICOS expression shown by representative flow cytometry plots of PBMCs gated on CD3^+^CD4^+^ from a healthy donor (HD) and members A.I.1, A.I.2, and A.II.1 (patient) of family A. Numbers in quadrant corners show population frequencies, additional numbers in upper right corner (top) represent mean fluorescence intensity (MFI) of PD-1 expressed by CXCR5^+^PD-1^+^ cells. **(B)** Flow cytometry plots of PBMCs showing PD-1 and TIGIT expression by CXCR5^+^CD4^+^ T cells. **(C)** Left panels: frequency of PD-1 and TIGIT expressing CXCR5^+^ and ICOS^+^ CD4^+^ T cells of HD (green), A.I.1 (orange), A.I.2 (red), and A.II.1 (red/orange). Middle panels: frequency of PD-1^+^ and of TIGIT^+^ CD4^+^ T cells and mean fluorescence intensity (MFI) of PD-1 and TIGIT expressed by CD4^+^ T cells of HD, A.I.1, A.I.2, and A.II.1 **(D)** PD-1 and TIGIT expression by an unrelated cytotoxic T lymphocyte antigen-4 (CTLA-4) haploinsufficiency patient with a heterozygous p.Y89X CTLA-4 mutation. **(E)** Frequency of PD-1 and TIGIT expressing CD4^+^ cells and expression levels of PD-1 and TIGIT determined by the MFI of PD-1 and TIGT expressed by CD4^+^ T cells treated for 5 days with or without (unst.) IL-7 and IL-15 in the presence or absence of the JAK inhibitor tofacitinib. Data in **(C)** represent mean ± SEM of 26 different donors (HD, CXCR5^+^, and PD-1^+^ICOS^+^) and two family visits, or mean ± SEM of 4 (TIGIT) and 7 (PD-1) different HDs and 2 (TIGIT) and 3 (PD-1) family visits, in **(D)** the mean ± SEM of five different HDs and the CTLA-4 p.Y89X patient, and in **(E)** show the mean ± SEM of three experiments with six different HDs. ****P ≤ 0.0001; ***P < 0.001; **P < 0.01; *P ≤ 0.05. One-way (**A,C**) or two-way **(D)** ANOVA, Dunnett’s multiple comparisons test.

Circulating PD-1^+^CD4^+^CXCR5^+^ T cells were increased in frequency with a large proportion of cells expressing ICOS (Figure 5A). Moreover, CXCR5^+^PD-1^+^ T cells of A.II.1 expressed more PD-1 than the cells of all controls (Figure 5A). Most of these CXCR5^+^ T_FH_ cells also expressed TIGIT (Figure 5B), a “checkpoint” molecule expressed by circulating T_FH_ cells. This subset was reported to have an increased capacity in supporting B cell proliferation and class switch recombination (25). In the patient, all CXCR5^+^TIGIT^+^ T cells were expressing PD-1 (Figure 5B). A slight, but significant increase of these activation markers was also observed on CD4^+^ T cells of the non-symptomatic CTLA-4 Y139C carrier A.I.1 compared to controls (Figure 5C). Since the phenotypic analysis of another patient with CTLA-4 haploinsufficiency (p.Y89X) but wild-type-*JAK3* identified in this study revealed normal expression levels of PD-1 and TIGIT in circulating CD4^+^T cells (Figure 5D), we tested if activation of the JAK3 pathway as found for the R840C mutation upregulates PD-1 and TIGIT expression in CD4^+^ T cells. To this end, we activated CD4^+^ T cells from healthy controls with IL-7 and IL-15 in the presence and absence of the JAK inhibitor tofacitinib. While activation with IL-7 and IL-15 lead to an increase in PD-1 and TIGIT surface expression, the upregulation was blocked by tofacitinib (Figure 5E). This suggests that the enhanced activity of the JAK3-R840C variant might contribute to the unusual PD-1^+^TIGIT^+^ T_FH_ cell phenotype of the patient.

### Reduced Cytokine Secretion Impairs T_FH_ Cell Function

**Figure 6:**
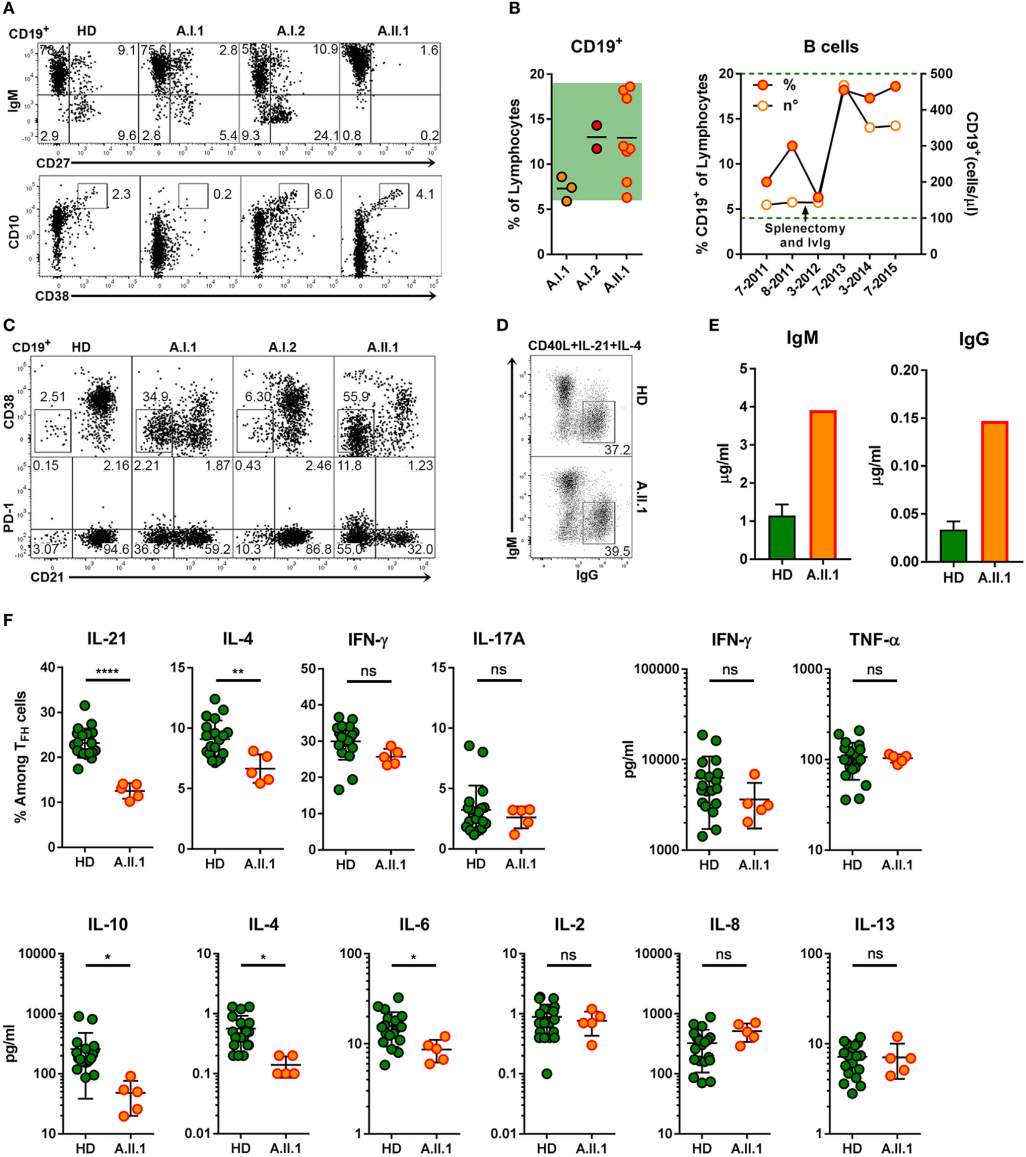
B cell phenotype, *in vitro* differentiation of B cells and impaired function of T_FH_ cells. **(A)** Representative flow cytometry plots of PBMCs stained for CD19, CD10, CD27, CD38, and IgM. The plots display CD19^+^ lymphocytes and show the population frequencies of transitional (CD10^+^CD38^++^), naïve (IgM^+^CD27^–^), marginal zone (IgM^+^CD27^+^), or switched memory (IgM^–^CD27^+^) B cell subsets. **(B)** Frequencies of CD19^+^ B cell from family members (left panel) and relative and absolute numbers of patient B cells within a 4-year period (right panel). Horizontal lines represent mean values. Each dot represents the mean of replicate assays and repeated experiments on different visits. Green background (left panel) and dotted line indicate normal range of B cell frequencies and of absolute numbers.**(C)** Representative flow cytometry plots of CD19^+^ lymphocytes from healthy donor (HD) and family members A.I.1, A.I.2, and A.II.1 (patient) stained for CD19, CD21, CD38, and PD-1. The numbers indicate population frequencies. Accumulation of CD38^−^CD21^lo^ (top) and of CD21^lo^PD-1^+^ (bottom) B cells in the patient (A.II.1) and the father (A.I.1) compared to a representative HD. **(D)** Frequencies of class-switched IgG^+^ B cells developing *in vitro* from IgM^+^IgG^−^ B cells activated with CD40L, IL-4, and IL-21 for 5 days. **(E)** IgM and IgG concentrations in the supernatants of CD19^+^CD27^−^IgG^−^IgA^−^ B cells activated *in vitro* for 6 days with CD40L, IL-4, and IL-21. **(F)** Left panels: frequency of IL-21, IL-4, IFN-γ, and IL-17A expressing CD4^+^ T cells after coculture with allogeneic CD19^+^CD27^−^IgG^−^IgA^−^ sorted B cells and re-stimulation with phorbol 12-myristate 13-acetate and ionomycin. Right and lower panels: cytokine concentrations in the supernatants of CD4^+^CD45RA^−^CXCR5^+^ sorted T_FH_ cells cultivated with CD19^+^CD27^−^IgG^−^IgA^−^ stimulator B cells from blood of five HD and from the patient. Data are representative for **(A,C,D)** or show mean ± SEM **(E)** of three different HDs and the patient (A.II.1) or mean ± SEM **(F)** of different B and T cell coculture combinations from five HD and the patient. Significant differences (two-tailed, unpaired *t*-test) between control subjects and patient are indicated. *P = 0.05; **P = 0.01, ****P < 0.0001.

Extrapolating from the observations made in CTLA-4 deficient mice (9), it would have been expected that the increased proportion of T_FH_ cells in patient A.II.1 enhances plasma cell development. However, consistent with the patient’s hypogammaglobulinemia, immunohistochemical analysis of LN sections revealed very few extrafollicular CD138^+^ plasma cells although IgG^+^ cells were detectable within the GCs (Figure 4A). Numbers of circulating B cells were normal but lacked a CD27^+^ memory B cell compartment (Figure 6A). Interestingly, B cell numbers increased and remained at relatively high counts after splenectomy without being enriched for CD10^+^CD38^+^ transitional B cells (Figures 6A,B). In addition, the compartment of circulating B cells contained mainly CD21^lo^ cells, which are likely to represent recently activated cells that respond poorly to new stimulating signals (Figure 6C) (26). An increase in CD21^lo^ B cells observed in HIV patients (27) is mainly attributed to the expansion of dysfunctional T-helper cells (28). To discriminate between intrinsic B cell defects impairing the differentiation into IgG switched memory B cells and plasma cells and disturbed T_FH_ cell function, we next tested *in vitro* differentiation of B cells and cytokine secretion by activated T cells.

The intrinsic B cell defect was excluded as CD27^−^IgG^−^IgA^−^ sorted (naïve) B cells of A.II.1 developed *in vitro* into class-switched IgG^+^ (Figure 6D) and antibody secreting cells (Figure 6E) similar to naïve B cells from healthy controls. Next, we tested T_FH_ cells function by monitoring cytokine production. The frequency of IL-21 and IL-4-expressing T_FH_ cells from A.II.1 and the amount of secreted IL-4, IL-6, and IL-10 were significantly lower, while other cytokines appeared normal compared to T_FH_ cells from HDs (Figure 6F). Thus, T_FH_ cells from A.II.1 are deficient in providing the key cytokines IL-21 and IL-4, which are crucial for the differentiation, expansion, class-switch recombination, and survival of antigen-selected B cells in the GC (29, 30). The molecular cause for the selectively impaired production of IL-21, IL-4, and IL-10 is not understood yet, but the high expression of PD-1, a negative regulator of T cell activation (31) might interfere with cytokine secretion and contribute to the immunodeficiency.

## Discussion

In our study, we found a novel heterozygous missense mutation in one allele of the CTLA-4 gene causing a non-synonymous amino acid exchange (p.Y139C) in the MYPPPY ligand-binding motif of the CTLA-4 protein. The mutation completely abrogates the binding of CTLA-4 to CD80 and CD86 and the ability of CTLA-4 Y139C expressing cells to suppress T cell proliferation *in vitro*. However, the patient’s father carries the same *CTLA4* mutation without showing any clinical symptoms. Therefore, lymphadenopathy, splenomegaly, low IgG concentrations, the absence of memory B cells, and the increase of the CD21^lo^ B cell subset found in the patient resemble an immune dysregulation syndrome resulting from CTLA-4 haploinsufficiency with incomplete penetrance, as it has been described before for other heterozygous mutations in *CTLA4* (5–7, 21, 32–34). Since the same *CTLA4* mutations are found in patients and in healthy carriers, additional, potentially genetic factors were postulated to contribute to the pathologic manifestations of the CTLA-4 haploinsufficiency-associated immunodysregulation syndrome (32–34).

To explain the incomplete penetrance of the heterozygous CTLA-4 mutation found in this family, we searched the exome of the mother (A.I.2) for heterozygous mutations with the potential of changing lymphocyte function, which were inherited by the patient (A.II.1) but absent in the exome of the father (A.I.1). Out of a set of 25 candidate genes, we identified a heterozygous missense mutation in *JAK3* changing the arginine residue R840 to cysteine. This residue forms a central part of the JAK3 protein, which is conserved in evolution from fish to humans. Located in a region sharing high homology to the JH2-JH1 domains of TYK2 (11), the R840 residue of JAK3 lies at the interface between the kinase domain and the pseudokinase JH2 domain. At this position, the arginine residue can form van-der-Waals contacts with I825 and contribute to the stabilization of the JH2-JH1 complex through H-bond interactions with F602/E552 or by ionic interactions with the side-chain of E552. The JAK3-R840C variant has been reported to have intrinsic kinase activity, as it was found to overcome resistance to a JAK1 inhibitor in an *in vitro* culture system (35). Interestingly, the gain-of-function mutation R867Q of JAK2 (36) is located at a position corresponding to amino acid 840 in JAK3 (11), suggesting a critical role of these residues in controlling the activity of JAK proteins. Similar to the acute myeloid leukemia associated gain-of-function variant JAK3-A572V (37), expression of the JAK3-R840C variant enhanced the proliferation of transduced primary T cells and of cell lines *in vitro*. Although the *in vitro* system does not fully reflect the more complex *in vivo* situation, it provides experimental evidence for a potential contribution of the JAK3-R840C variant to the pronounced lymphoproliferation in A.II.1 with underlying CTLA-4 syndrome. The clinically unremarkable phenotype of the mother, who is a JAK3 R840C carrier, suggests that the R840C missense mutation still allows normal T cell homeostasis in the presence of normal CTLA-4 function. The fact that the JAK3-R840C variant has so far been detected only in an *in vitro* selection system (35), but never in conjunction with malignancy or lymphoproliferative disease supports our reasoning.

The lowered activation threshold *via* the JAK-STAT pathway caused by the R840C mutation combined with insufficient control of activated T cells caused by the CTLA4 Y139C haploinsufficiency may also account for the high proportion of circulating CD4^+^PD-1^+^TIGIT^+^ T cells. This T cell population includes a significant fraction of circulating CXCR5^+^ICOS^+^PD-1^+^ T_FH_ cells expressing very high levels of PD-1, which might be caused by the intrinsic kinase activity of the JAK3 R840C variant.

The skewing of the T cell compartment toward PD-1^+^TIGIT^+^ T_FH_ cells might give rise to the highly enlarged germinal centers observed in the LN and the spleen. PD-1, in particular, is essential for a sustained GC reaction (31). In addition, we detected in the follicles a set of relevant markers like BCL-6, ICOS, IRF-4, and IgG, which are indicative of GC activity. In spite of the enlarged T_FH_ cell compartment, circulating switched memory B cells were missing and the output of CD138^+^ plasma cells from germinal centers was found to be severely impaired leading to hypogammaglobulinemia. This paradoxical immunophenotype of an expanded T_FH_ cell compartment with large follicles but defective late B cell differentiation has similarities to the impaired B cell immunity observed in HIV-infected individuals (38). In HIV-infected individuals, impaired vaccination responses in the presence of large lymphoid follicles in the LNs has been attributed to the reduction in IL-21 secretion by T_FH_ cells caused by interactions between PD-L1 and PD-1, expressed by germinal center B cells and by T_FH_ cells, respectively. Our data fit well into this scenario, because *in vitro* activated circulating T_FH_ cells of A.II.1 produced less IL-21 and IL-4 than T_FH_ cells from healthy controls. In contrast to the disturbed T_H_2 activity, IFN-γ and IL-17 production was normal. Since IL-21 and IL-4 are key GC cytokines driving B cell proliferation and differentiation into plasma cells (29, 30), the PD-1^+^TIGIT^+^IL-21^lo^IL-4^lo^ T_FH_ cells may not provide sufficient levels of cytokines to support the development of GC B cells into normal plasma cells. In addition to PD-1, the co-expression of the negative regulator TIGIT by CD4^+^PD-1^+^ T cells may contribute to the inhibition of T cell function, as reported for T cells treated with agonistic anti-TIGIT antibodies (39).

Therefore, the development of dysfunctional T_FH_ cells offers an explanation for the almost complete absence of plasma cells in the LN and hypogammaglobulinemia despite high GC activity in our patient.

In summary, we report here that a rare genetic variant of *JAK3* may change a clinically unremarkable CTLA-4 haploinsufficiency to an overt CTLA-4 syndrome. However, the combination of mutations activating JAK3 with *CTLA4*-inactivating mutations seems to be very rare since the screening of 52 patients with CTLA-4 haploinsufficiency did not reveal another case with a missense mutation in the JAK3 gene. Therefore, also other factors including genetic modifiers can determine the clinical penetrance of mutations in the CTLA-4 gene.

Other clinically related syndromes may result from the combination of hypomorphic/hypermorphic, heterozygous mutations, in genes regulating the immune system. For example, incomplete penetrance, exemplified by a significant fraction of non-symptomatic carriers, has been described for genetic defects associated with lymphoproliferation and lymphadenopathy, such as activated PI3Kδ syndrome (APDS) (40–43) or *FAS-*associated autoimmune lymphoproliferative syndrome (ALPS-FAS) (44). In general, hyperimmunity and lymphoid hyperplasia or autoimmunity is not uncommon in patients with primary antibody deficiency (45, 46), and an aberrant T_FH_ phenotype similar to the one we describe here may underlie this paradox. Our findings may stimulate the search for combinations of immune-modulating gene variants in CVID patients to identify pathogenic molecular and cellular pathways and to potentially enable individualized and targeted therapy.

## Ethics Statement

This study was approved and carried out in accordance with the recommendations of the Ethics Committee of the University of Freiburg; with written informed consent from all subjects. All subjects gave written informed consent in accordance with the Declaration of Helsinki. The protocol was approved by the Ethics Commission of the University of Freiburg.

## Author Contributions

HS, MS, ET, HG, and HE designed experiments. HS performed most experiments and analyzed data. MS, and ES contributed human samples and performed DNA sequence analyses. VC, MS, and ES performed experiments. EO performed whole exome sequencing and MB, BL, and FY contributed bioinformatics analysis of exome data. EV contributed protein modeling. AG, AS, ET, HG, and HE provided reagents and intellectual input. HS, HG, and HE wrote the manuscript with input from AS, MS, and ET All authors discussed and revised the manuscript.

## Conflict of Interest Statement

HS, VC, MB, FY, EO, EV, AS, ET, and HG are employees of Novartis Pharma AG, Basel, Switzerland. All other authors declare no potential conflict of interest.
