# Supplementary material for: An Activating Janus Kinase-3 Mutation Is Associated with Cytotoxic T Lymphocyte Antigen-4-Dependent Immune Dysregulation Syndrome
Source: Front Immunol. 2017 Dec 15;8:1824. doi: 10.3389/fimmu.2017.01824 (PMC5770691; doi:10.3389/fimmu.2017.01824)
Supplement: Supplementary file 1 [file Data_Sheet_1.docx]

Supplementary Material

An activating JAK3 mutation is associated with CTLA-4-dependent immune dysregulation syndrome.

**Authors:** Heiko Sic^1^, Matthaios Speletas^2^, Vanessa Cornacchione^1^, Maximillian Seidl^3,5^, Martin Beibel^1^, Bolan Linghu^4,§^, Fan Yang^4^, Eirini Sevdali^2^, Anastasios E. Germenis^2^, Edward J. Oakeley^1^, Eric Vangrevelinghe^1^, Andreas W. Sailer^1^, , Elisabetta Traggiai^1^, Hermann Gram^1^, Hermann Eibel^5*^

*** Correspondence:** Corresponding Author hermann.eibel@uniklinik-freiburg.de

**Supplementary Data**

**1 Supplementary methods.**

1.1 Library preparation, DNA sequencing, data alignment and analysis

Exon-specific next-generation sequencing was performed similar to Kaufman et al. (1) using ds-DNA sheared an average size of 300 bp. After construction, amplification, and size selection each library was uniquely indexed, captured and sequenced. Index demultiplexing was performed using an Illumina CASAVA suite (San Diego, USA), and read quality was controlled with the FASTQC package (Brabham Inst., Cambridge, UK). Alignment and variant calls were made with the Broad Institute’s Genome Analysis Toolkit. Reads that passed Illumina’s Chastity Filter were aligned to the human genome reference HG19. All reads overlapping with known or putative indels were realigned. Base quality scores were recalibrated to the empirical error rate derived from nonpolymorphic sites. Single nucleotide variants and small INDELs were called in all samples simultaneously using the UnifiedGenotyper module in Genome Analysis Toolkit (v1.6-13-g91f02df). Variants were annotated with VEP software version 2.5 and Ensembl data base homo_sapiens version 67. Tabular data and VEP output were merged and processed with custom R scripts (under R version 2.15.2). Each variant was annotated by its most severe consequence according to Polyphen and Sift predictions and ranked according as proposed in http://www.ensembl.org/info/genome/variation/predicted_data.html. The resulting data table was then loaded into Excel.

1.2 FACS analysis

For phenotyping and intracellular CTLA-4 and FOXP3 staining 0.3-1x10^6^ PBMCs were processed directly *ex vivo* or activated overnight with anti-CD3 (1 µg/ml, OKT-3, Novartis, Basel, Switzerland) and anti-CD28 (1 µg/ml, 15E8, Novartis) in complete IMDM. Cells were washed with PBS, stained for viability (Zombie Yellow, Bio-legend, San Diego, USA, 10 min, RT, in the dark), washed with cold FACS buffer (PBS, 3% FCS, 0.1% NaN_3_) and incubated (20 min, 4° C, in the dark) with 50 μl FACS buffer containing different combinations of antibodies against surface antigens CD3 (OKT-3), CD10 (HI10a), CD38 (HIT2), CD45RA (HI100), IgM (MHM-88), CCR7 (G043H7), PD-1 (EH12.2H7, all Biolegend), CD19 (SJ25-C1, SouthernBiotech, Birmingham, USA), CD4 (RPA-T4), CD8 (RPA-T8), CD25 (M-A251), CD27 (M-T271), CD127 (HIL-7R-M21), IgD (IA6-2, all BD Biosciences, Franklin Lakes, USA), CD21 (HB5), CXCR5 (MU5UBEE), ICOS (ISA-3), TIGIT (MBSA43, all eBioscience, San Diego, USA), IgA (109-096-011, Jackson ImmunoResearch, West Grove, USA) and IgG (F031501, Dako), or appropriate isotype control antibodies. Cells were then washed and analyzed, or fixed and permeabilized using the FOXP3 staining kit (eBioscience) following the manufacturer’s instructions, stained for FOXP3 (236A/E7, eBioscience) and CTLA-4 (BNI3, BD), and assessed using an LSRFortessa (BD) flow cytometer and FlowJo software (Tree-Star, Ashland, USA).

Basal pSTAT5 levels were assessed by intracellular staining after 24 h incubation of infected primary CD4 T cells in normal culture medium. 3x10^5^ cells in 200 µl normal culture medium were fixed by addition of 2 ml pre-warmed Lyse/Fix-buffer (BD, 20 min, 37° C). Cells were then washed and permeabilized with Methanol (90%, 1 h, -20° C). After washing cells were stained with 50 μl FACS buffer containing antibodies against CD4 (RPA-T4, Biolegend), CD25 (M-A251, BD) and pSTAT5 (47/Stat5(pY694), BD).

1.3 Histology

Formalin-fixed and paraffin embedded sections were stained after deparaffinization and antigen retrieval with anti-IgD (rabbit polyclonal, IS517), Ki67 (MIB-1), BCL-6 (PG-P6p), IRF-4 (MUM1p), IgG (rabbit polyclonal, IS512), IgM (rabbit polyclonal, IR513), CD4 (4B12), CD20 (L26), CD138 (MI15, all Dako, Glostrup, Denmark), PD-1 (AF1086, R&D Systems, Minneapolis, USA), CD25 (4C9, Novocastra/Leicabiosystems, Nussloch, Germany), PD-L1 (E1L3N, Cell Signaling, Danvers, USA) and BLIMP-1(3H2-E8, Novus Biologicals; Littleton, USA).

1.4 Western blotting

Phosphorylation of STAT5 was analyzed by western blotting using anti-phospho-STAT5 (D47E7), anti-STAT5, anti-JAK3 (D44E3, all Cell Signaling) and anti-β-Actin (Sigma-Aldrich) antibodies.

**2. Supplementary Figures.**

**2.1 Supplementary Figure 1.**

**
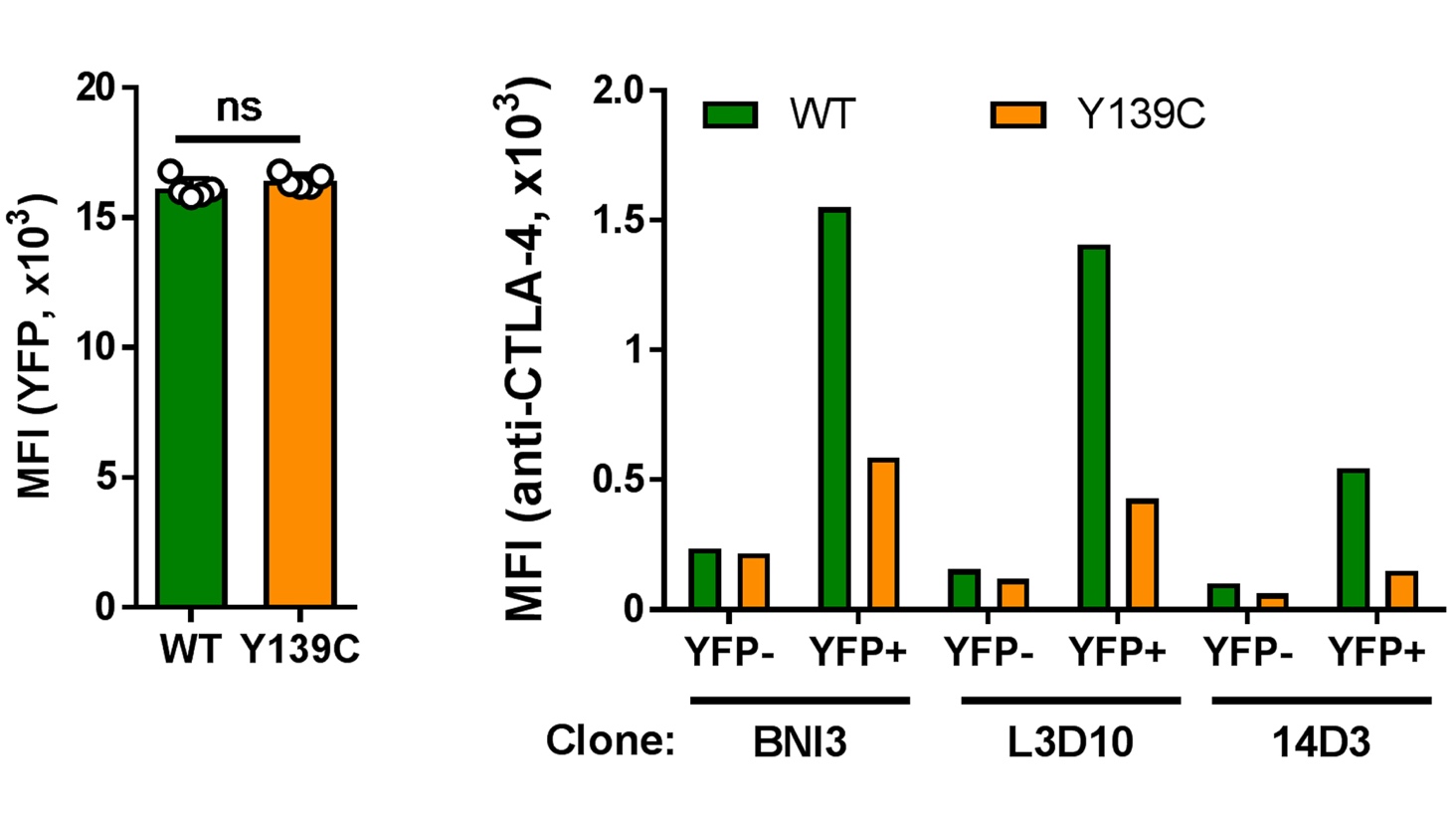
**

**Total and cell surface expression levels of wildtype and Y139C mutant CTLA-4.** Jurkat T cells were transduced with lentiviral expression vectors encoding wildtype or Y139C mutant CTLA-4 proteins fused at their C-terminal end to YFP and analyzed by flow cytometry. Left panel: total CTLA-4-YFP expression levels for the wildtype (green) and the Y139C mutant protein were determined by comparing the mean fluorescence intensities (MFI) of YFP. Right panel: To determine cell surface expression, a mixture of transduced and untransduced Jurkat T cells was stained with three different, APC-conjugated anti-CTLA-4 antibodies (BNI3, Becton Dickinson; L3D10, Biolegend; 14D3, Thermofisher) and analyzed by flow cytometry for total CTLA-4 expression by recording the YFP signal and for CTLA-4 cell surface expression by recording the signal of the APC-labelled anti-CTLA-4 antibodies. The plot displays the mean fluorescence intensity (MFI) of surface wildtype and Y139C mutant CTLA-4 on transduced (YFP+) and untransduced cells (YFP-)

**2.2 Supplementary Figure 2.**


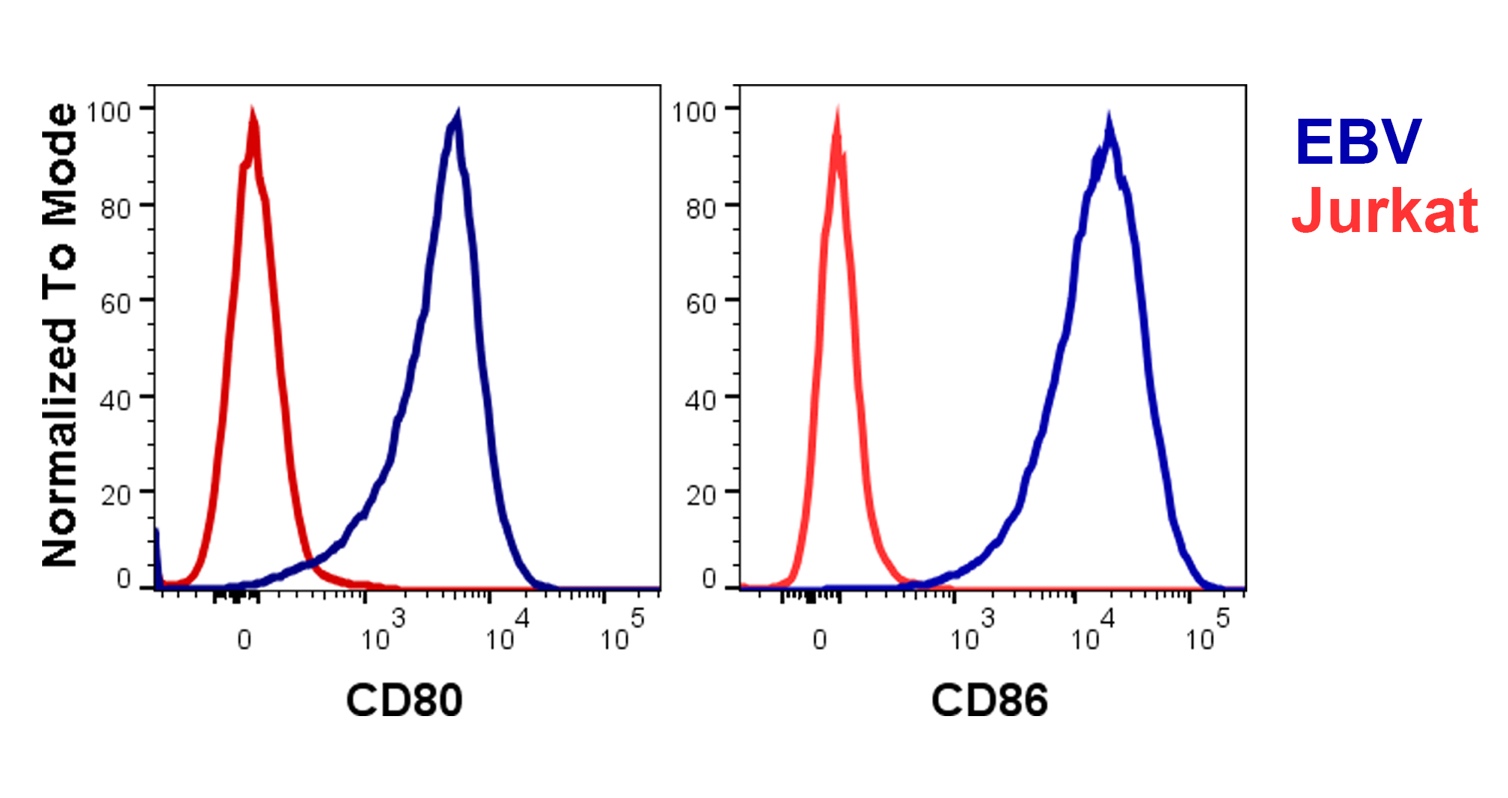


**Cell surface expression levels of CD80 and of CD86 on EBV cells** used as stimulator cells in T cell activation assays. CD80. Cells were stained with anti-CD80 (2D10 coupled to Alexa 647; Biolegend) and anti-CD86 (IT.2., coupled to Alexa 647; Biolegend) and analyzed by flow cytometry. Jurkat T cells used in the same assays as host cells for the expression of wildtype and Y139C mutant CLTA-4 served as control.

**2.3 Supplementary Figure 3.**

**Suppression of T cell proliferation by regulatory T cells.** Naïve CD4^+^ responder T (T_RESP_) cells were labelled with the Cell Proliferation Dye eFluor450 (Thermofisher) and activated with anti-CD3 in presence of heterologous EBV cells. T cell proliferation was inhibited by adding sorted CD4^+^CD25^+^IL7R^-^ T_REG_ cells from healthy donors or from family A members A.I.1, A.I.2 or A.II.1 (patient). Ratios 5:1:5 (T_RESP_:EBV:T_REG_). The cell suspensions were then incubated for 4 days and analyzed by flow cytometry for the proliferation of labeled CD4^+^ T_RESP_ cells. In contrast to the T_REG_ cells of the patient A.II.1, T_REG_ cells of healthy donors and of family members A.I.1 and A.I.2 suppress the proliferation of responder T cells. The suppression of responder T cell proliferation by the T_REG_ cells of asymptomatic *CTLA4* mutation carriers has been descried before (2). Except for the sample A.I.1, the assays were carried out with two different responder T cell samples and T_REG_ cells from two different healthy donors in duplicates or triplicates whenever possible.

**3. Supplementary Tables**

**3.1 Supplementary Table 1.**

| **Chr** | **Gene** | **Consequence** | **Annotated variation** | **GMAF** | **POS** | **REF** | **ALT** | **A.I.1** | **A.I.2** | **A.II.1** | **A.II.2** |
| --- | --- | --- | --- | --- | --- | --- | --- | --- | --- | --- | --- |
| 1 | *CLSTN1* | missense | rs35331030 | T:0.0046 | 9811653 | C | T | 0 | 1 | 1 | 0 |
| 1 | *EFCAB14* | missense | - |  | 47183702 | T | C | 0 | 1 | 1 | 1 |
| 2 | *SPTBN1* | missense | rs200348448, COSM1021721 | A:0.0009 | 54895587 | G | A | 0 | 1 | 1 | 1 |
| 3 | *CCR5* | missense | rs145061115 |  | 46414451 | T | A | 0 | 1 | 1 | 0 |
| 4 | *N4BP2* | missense | - |  | 40113716 | T | A | 0 | 1 | 1 | 1 |
| 4 | *POLR2B* | missense | - |  | 57877207 | G | A | 0 | 1 | 1 | 0 |
| 5 | *FAM193B* | missense | - |  | 176963461 | G | A | 0 | 1 | 1 | 1 |
| 5 | *NDUFS6* | missense | rs77687671 | G:0.0023 | 1814739 | A | G | 0 | 1 | 1 | 0 |
| 5 | *TGFBI* | missense, NMD transcript | rs34334509 | T:0.0096 | 135385172 | C | T | 0 | 1 | 1 | 0 |
| 6 | *KCTD20* | missense | - |  | 36454726 | A | G | 0 | 1 | 1 | 0 |
| 6 | *SCML4* | missense | - |  | 108093476 | G | A | 0 | 1 | 1 | 1 |
| 6 | *RB1CC1* | missense | TMP_ESP_8_53555119 |  | 53555119 | G | A | 0 | 1 | 1 | 1 |
| 8 | *UTP23* | missense | - |  | 117778991 | C | G | 0 | 1 | 1 | 1 |
| 9 | *RABL6* | missense | - |  | 139735400 | G | A | 0 | 1 | 1 | 0 |
| 12 | *MMP19* | missense | rs150724096 | T:0.0027 | 56230980 | C | T | 0 | 1 | 1 | 0 |
| 12 | *SLC15A4* | missense | rs144816528 | A:0.0005 | 129299599 | G | A | 0 | 1 | 1 | 0 |
| 14 | *NUBPL* | missense | TMP_ESP_14_32295853 |  | 32295853 | C | T | 0 | 1 | 1 | 0 |
| 15 | *NPAP1* | missense | rs146375975 | A:0.0018 | 24924316 | G | A | 0 | 1 | 1 | 1 |
| 16 | *IGFALS* | missense | - |  | 1842263 | G | A | 0 | 1 | 1 | 0 |
| 16 | *SH2B1* | missense | - |  | 28884870 | C | T | 0 | 1 | 1 | 1 |
| 19 | *EMC10* | missense | - |  | 50983428 | G | A | 0 | 1 | 1 | 0 |
| 19 | *JAK3* | missense | rs200077579 |  | 17943490 | G | A | 0 | 1 | 1 | 0 |
| 19 | *SAE1* | missense | rs199801330 |  | 47646779 | G | A | 0 | 1 | 1 | 0 |
| 21 | *BRWD1* | missense, NMD transcript | - |  | 40630521 | G | A | 0 | 1 | 1 | 1 |
| X | *RLIM* | missense | - |  | 73812299 | C | T | 0 | 1 | 1 | 0 |

**Rare single nucleotide polymorphisms shared by A.I.2 and AII.1 but not by AI.1.**

To detect potential modifiers of the *CTLA4* mutation found in the exomes of A.I.2 and A.II.1 but not in A.I.1, the SNPs of all family A members were compared. The table shows a list of candidate genes carrying potentially deleterious mutations fulfilling the selection criteria. Table legend: missense = missense variant; NMD transcript = mutation has the potential to induce nonsense-mediated decay of the transcript; POS = chromosomal position. REF = reference allele; ALT = changed allele; 0 = absent; 1 = present.

**References.**

1. Kaufman KM, Linghu B, Szustakowski JD, Husami A, Yang F, Zhang K, Filipovich AH, Fall N, Harley JB, Nirmala NR, et al. Whole-Exome Sequencing Reveals Overlap Between Macrophage Activation Syndrome in Systemic Juvenile Idiopathic Arthritis and Familial Hemophagocytic Lymphohistiocytosis. *Arthritis Rheumatol* (2014) **66**:3486–3495. doi:10.1002/art.38793

2. Kucuk ZY, Charbonnier L-M, McMasters RL, Talal Chatila M, Bleesing JJ. CTLA-4 haploinsufficiency in a patient with an autoimmune lymphoproliferative disorder. (2017) doi:10.1016/j.jaci.2017.02.032
